# Impairments of working memory in schizophrenia and bipolar disorder: the effect of history of psychotic symptoms and different aspects of cognitive task demands

**DOI:** 10.3389/fnbeh.2014.00416

**Published:** 2014-11-28

**Authors:** Dorota Frydecka, Abeer M. Eissa, Doaa H. Hewedi, Manal Ali, Jarosław Drapała, Błażej Misiak, Ewa Kłosińska, Joseph R. Phillips, Ahmed A. Moustafa

**Affiliations:** ^1^Department and Clinic of Psychiatry, Wroclaw Medical UniversityWroclaw, Poland; ^2^Faculty of Medicine, Psychogeriatric Research Center, Institute of Psychiatry, Ain Shams UniversityCairo, Egypt; ^3^Institute of Computer Science, Wroclaw University of TechnologyWroclaw, Poland; ^4^Department of Genetics, Wroclaw Medical UniversityWroclaw, Poland; ^5^School of Social Sciences and Psychology, Marcs Institute for Brain and Behaviour, University of Western SydneySydney, NSW, Australia

**Keywords:** schizophrenia, psychotic vs. non-psychotic bipolar disorder, working memory, history of psychosis

## Abstract

Comparisons of cognitive impairments between schizophrenia (SZ) and bipolar disorder (BPD) have produced mixed results. We applied different working memory (WM) measures (Digit Span Forward and Backward, Short-delay and Long-delay CPT-AX, N-back) to patients with SZ (*n* = 23), psychotic BPD (*n* = 19) and non-psychotic BPD (*n* = 24), as well as to healthy controls (HC) (*n* = 18) in order to compare the level of WM impairments across the groups. With respect to the less demanding WM measures (Digit Span Forward and Backward, Short-delay CPT-AX), there were no between group differences in cognitive performance; however, with respect to the more demanding WM measures (Long-delay CPT-AX, N-back), we observed that the groups with psychosis (SZ, psychotic BPD) did not differ from one another, but performed poorer than the group without a history of psychosis (non-psychotic BPD). A history of psychotic symptoms may influence cognitive performance with respect to WM delay and load effects as measured by Long-delay CPT-AX and N-back tests, respectively. We observed a positive correlation of WM performance with antipsychotic treatment and a negative correlation with depressive symptoms in BPD and with negative symptoms in SZ subgroup. Our study suggests that WM dysfunctions are more closely related to a history of psychosis than to the diagnostic categories of SZ and BPD described by psychiatric classification systems.

## Introduction

The dichotomy between mood disorders and schizophrenia has been described by Kraepelin in the nineteenth century, mainly based on their different illness course and prognosis (Kraepelin, 1921). However, until today the nosological and clinical implications of psychotic features in the course of mood disorders (Henry and Etain, 2010) and the concept of unitary psychosis including affective and non-affective psychotic disorders are widely debated (van Os et al., 2000; Angst, 2002; Peralta and Cuesta, 2005; Reininghaus et al., 2013). Current diagnostic systems make a categorical distinction between the disorders that are primarily affective and the disorders that are primarily psychotic in nature. However, psychotic and affective disorders are increasingly perceived as dimensionally different rather than categorically separate entities (Kane and Engle, 2002). This concept is supported by research showing that neuropsychological dysfunctions among psychotic and affective disorders depend on a history of psychosis rather than a diagnostic group itself.

Neurocognitive dysfunctions are core characteristics of schizophrenia (SZ) and are increasingly recognized as an important feature of bipolar disorder (BPD) (Vohringer et al., 2013). However, comparisons of cognitive impairments between BPD and SZ have produced mixed results. Some authors indicate that patients with BPD exhibit a similar pattern of deficits to patients with SZ, but the level of impairment can be placed between SZ patients and healthy controls (HC) (Krabbendam et al., 2005; Daban et al., 2006; Schretlen et al., 2007; Hamilton et al., 2009; Reichenberg et al., 2009), while others show no qualitative or quantitative neurocognitive differences between patients with SZ and BPD (Mojtabai et al., 2000; McClellan et al., 2004; Simonsen et al., 2011).

It has been suggested that the discrepancy in these findings may be explained by the fact that neurocognitive dysfunction in BPD and SZ spectrum disorders depend on a history of psychosis rather than a diagnostic group (Simonsen et al., 2011; Ivleva et al., 2012). (Pearlson et al., 1995; Strasser et al., 2005; Glahn et al., 2007; Cui et al., 2011; Anticevic et al., 2014). This hypothesis has been supported by findings showing that BPD patients with psychotic symptoms [BPD(+)] perform worse than BPD patients without psychotic symptoms [BPD(−)] on a variety of neurocognitive measures (Glahn et al., 2006, 2007; Bora et al., 2007; Martinez-Aran et al., 2008; Allen et al., 2010; Simonsen et al., 2011; Ivleva et al., 2012). In the recent meta-analytic study, a history of psychotic symptoms in BPD was found to be associated with moderately greater impairment in planning and reasoning, working memory, verbal memory and processing speed (Bora et al., 2010). Interestingly, it has been suggested that unlike BPD(−), BPD(+) is associated with biological correlates typical for SZ (Glahn et al., 2007), such as significantly enlarged lateral and third ventricles (Strasser et al., 2005), increased D2 dopamine receptor density (Pearlson et al., 1995), reduced neural connectivity (Anticevic et al., 2014) and gray matter deficits (Cui et al., 2011).

Working memory (WM) is the system for the temporary storage of information on a moment-to-moment basis and is a critical function that underlies various complex cognitive tasks such as language, comprehension, learning and reasoning (Baddeley, 1992). WM is a complex and multifaceted construct. It is able to encode, retrieve, store, update, and manipulate information in the mental workspace. Strong evidence has accrued that these processes are relatively separate (Cornoldi et al., 2000; Woodman and Vogel, 2005) and that there is high individual variability in their capacity (Kane and Engle, 2002). Neuroimaging studies provide evidence that these processes may also involve different neural networks (Jolles et al., 2011; Marvel and Desmond, 2012; Liao et al., 2014) and are associated with different neural network activity level (Veltman et al., 2003; Jolles et al., 2011). The capacity of different WM processes can be assessed by tasks that manipulate either the load of data to be stored (load effect), the duration of the maintenance period (delay effect) or the number of items to be manipulated within WM (manipulation effect) (Jolles et al., 2011; Pinal et al., 2014).

Although WM impairments were previously probed in SZ and BPD, comparisons across disorders have yielded inconsistent findings and the relationships between cognitive and clinical symptom domains across the psychotic variations of the disorders remain unclear (Hamilton et al., 2009; Mayer and Park, 2012; Milanovic and Vangel, 2012; Zhang et al., 2012). Discrepancy in previous studies may be due to a heterogeneous patient group and/or differences in specific task demands of the cognitive measures employed. Among other cognitive domains, WM has been shown to be associated with a history of psychosis rather than affective or psychotic diagnostic category by numerous studies (Bora et al., 2007; Glahn et al., 2007; Martinez-Aran et al., 2008; Savitz et al., 2009; Simonsen et al., 2011). However, WM tasks employed in the previous studies measure different components of WM, not allowing for comparisons across different levels of task demands (load of information, duration, or maintenance, number of items to be manipulated within WM). The lack of capacity measurements for the different WM domains make it impossible to precisely and reliably compare cognitive performance between different psychiatric subpopulations.

Taking into account the limitations of previous studies, the aim of our research is to examine different WM aspects in a cross diagnostic sample to determine as to whether the symptomatology dimension in terms of psychotic, affective and both psychotic and affective symptoms is a common denominator of different WM impairments in SZ and BPD patients. On the basis of the psychosis dimension concept, we hypothesize that BPD(+) and SZ patients share common WM deficits that are different than those observed in BPD(−) patients and that these groups of patients can be classified on the basis of WM performance. We assess three components of WM: the delay effect, measured with Short-delay and Long-delay CPT-AX tasks; the load effect, as measured using the Forward Digit Span (FDS) task and N-back task; and manipulation of information capacity was measured with the Backward Digit Span (BDS). Although there are many studies of WM in SZ and BPD, this is the only study to assess different aspects of WM with respect to psychosis dimension in these patient populations.

## Materials and methods

### Participants

We included 66 patients [24 BPD(−), 19 BPD(+) and 23 SZ] and 18 healthy controls (HC) in the study (for raw data see Supplementary Material 1). All HC were recruited from the community via ads, word of mouth, or others who participated in previous studies. Patients were recruited from the Psychogeriatric Research Center, Institute of Psychiatry, Faculty of Medicine, Ain Shams University, Cairo, Egypt. Diagnosis of BPD and SZ was based on Structured Clinical Interview for DSM-IV Axis I disorders (SCID-I) performed by the same trained clinician. Clinical data in SCID-I was gathered from personal interviews, clinical observation, medical records (hospital and outpatient clinic case notes) and family interviews (if the patient was unable to provide required information). Any patients with other schizophrenia spectrum disorders including: schizophreniform disorder, schizoaffective disorder, delusional disorder, brief psychotic disorder, substance-induced psychosis, and psychotic disorders due to general medical conditions, as well as first-episode psychosis of any type were not included in the study. None of the diagnoses were changed from those originally given by the treating psychiatrist. Patients diagnosed with BPD and had experienced delusions or hallucinations during a mood episode were included in the BPD(+) group. While BPD patients who had not experienced hallucinations or delusions during any of the mood episodes were included in the BPD(−) group. Psychotic symptoms were coded based on module B of SCID-I.

All patients were outpatients and had been on a stable medication regimen for at least 4 weeks prior to testing. All participants had no history of significant neurological injury and reported no serious medical illness or substance use disorders. None of the participants had any first-degree relatives with psychiatric illness. The demographic and clinical characteristics are shown in Tables 1, 2. All participants provided informed consent for a protocol approved by the Bioethical Committee of Ain Shams University and its associated clinics.

**Table 1:** **Demographic and clinical characteristics for the participants**.

	**HC**	**BPD(−)**	**BPD(+)**	**SZ**	*P*-value^*^
Number of participants	18	24	19	23	
Age (mean ± *SD*)	44.34 ± 6.99	43.01 ± 5.11	44.63 ± 4.02	42.09 ± 6.02	0.41
%Male	33.4	41.7	36.8	39.1	0.95
Education^a^ (mean ± *SD*)	11.94 ± 2.88	11.9 ± 2.96	11.63 ± 3.32	12.00 ± 3.00	0.97
NAART^b^ (mean ± *SD*)	105.76 ± 7.25	100.88 ± 9.14	104.72 ± 10.35	104.94 ± 8.55	0.98
CPZ^c^ equivalent dosage (mean ± *SD*)	–	403.54 ± 187.67	396.00 ± 127.6	373.00 ± 111.77	0.59
Duration of illness (mean ± *SD*)	–	18.29 ± 4.07	19.37 ± 4.64	17.52 ± 4.86	0.37
PANSS^d^ (mean ± *SD*)					
PANSS Negative Symptoms	–	–	–	19.35 ± 4.69	
PANSS Positive Symptoms	–	–	–	16.70 ± 3.98	
PANSS General Symptoms	–	–	–	24.48 ± 5.13	
YMRS^e^ (mean ± *SD*)	–	18.42 ± 3.97	24.00 ± 4.55	–	0.00
HDRS^f^ (mean ± *SD*)	–	23.17 ± 3.96	23.74 ± 2.99	–	0.30
AES^g^ (mean ± *SD*)	39.94 ± 5.33	36.20 ± 7.85	36.31 ± 7.61	35.88 ± 8.76	0.34

HC, healthy controls; BPD(−), BPD patients without history of psychosis; BPD(+), BPD patients with history of psychosis; SZ, patients with schizophrenia.

* p-value for Kruskal-Wallis test for continuous variables and for chi-square test for categorical variables.

a Number of years of completed education.

b Estimate of premorbid IQ measured by North American Adult Reading Test (NAART).

c CPZ, chlorpromazine.

d PANSS, Positive and Negative Symptoms Scale.

e YMRS, Young Mania Rating Scale.

f HDRS, Hamilton Depression Rating Scale.

g AES, Apathy Evaluation Scale.

**Table 2:** **Detailed information about treatment regime with respect to different patient groups**.

**Antipsychotic medication**	**BPD(−)**	**BPD(+)**	**SZ**
%Chlorpromazine only	12.5	26.3	13.0
%Clozapine only	8.3	52.6	47.8
%Haloperidol only	16.7	5.3	8.7
%Risperidone only	33.3	10.5	8.7
%Chlorpromazine plus another antipsychotic	8.3	5.3	8.7
%Clozapine plus another antipsychotic	12.5	–	4.3
%Haloperidol plus another antipsychotic	4.2	–	8.7
%Risperidone plus another antipsychotic	4.2	–	–

HC, healthy controls; SZ, schizophrenia; BPD(−), bipolar disorder without history of psychosis; BPD (+), bipolar disorder with history of psychosis.

### Materials

The participants of the study were assessed using a diagnostic interview, clinical scales and neuropsychological tasks. SZ patients were evaluated on Positive and Negative Syndrome Scale (PANSS) (Kay et al., 1987), BPD patients were assessed for depressive symptoms on Hamilton Depression Rating Scale (HDRS) (Hamilton, 1960) and for manic symptoms on Young Mania Rating Scale (YMRS) (Young et al., 1978). All participants were evaluated on Apathy Evaluation Scale (Marin et al., 1991). Premorbid IQ was estimated based on the performance on the North American Adult Reading Test (NAART) (Blair and Spreen, 1989). Neuropsychological tasks included Forward Digit Span (FDS) and Backward Digit Span (BDS) (Wechsler, 1997), as well as Short-delay CPT-AX, Long-delay CPT-AX and N-back task.

### Tasks

The experimenters were present during the entire testing session to ensure participants responded to all stimuli presentations.

#### Forward digit-span (FDS) and backward digit-span (BDS)

Digit Span tasks are used to measure WM's load capacity. Participants are presented with a series of digits and must repeat them back. If they do this successfully, they are given a longer list. The length of the longest list is the person's digit span. We used the Digit Span subtests of the Wechsler Adult Intelligence Scale III (WAIS-R-III) (Wechsler, 1997). Participants are required to recall the digit lists both in forward chronological order (FDS), and in reverse order (BDS).

#### Short-delay CPT-AX and long-delay CPT-AX

The CPT-AX task used here is a variation of delayed-response task. For similar designs see (Barch et al., 1997; Cohen et al., 1999; Moustafa et al., 2008; Paxton et al., 2008). Participants were presented with a sequence of four-letter stimuli, one at a time (H, K, Z, P). Participants were presented with H before Z, H before P, K before Z and K before P. Participants were instructed to figure out the target sequence by trial and error based on correct or incorrect feedback given to them after each stimulus. In this task, a correct response to a given letter depends on the letter preceding it. In the task, HZ is a target sequence and all other sequences (HP, KZ, KP) are incorrect.

Participants were to press a key on the left side of the keyboard (“z”) in case of non-target sequence, and a key on the right side of the keyboard (“m”) in case of target sequence. Correct and incorrect feedback were shown on the screen as green “CORRECT” or red “INCORRECT,” following participants' responses on each trial.

The instructions here were as follows: “In this task, you will have to figure out the target sequence by trial and error. At the end of each trial, you will get feedback to see if you were CORRECT or INCORRECT. You will use this feedback to figure out the right sequence. You will see the letters H, K, Z, P. Try to keep track of the letters. Press “z” button for every letter, EXCEPT when you think you have seen the target sequence, press “m” button. At first you will have to guess. You will figure out what the target sequence is as you get CORRECT/INCORRECT feedback to your button presses.”

The measure reported in this task is accuracy (percentage of correct responses) across all four trial types (HZ, HP, KZ, KP). There were 150 trials in total in the task. In Short-delay CPT-AX task, delay interval between each letter presentation was 1 s, while in Long-delay CPT-AX task, delay interval was 5 s and different set of letters was used (M, T, R, S).

#### N-back task

The N-back task tests the effects of WM load on performance (Cohen et al., 1997; Owen et al., 2005; Wallwork et al., 2012). In this task, a sequence of letters was presented to the participants, one at a time. The task involves the presentation of the sequences made up of the following distinct letters: BFKHMQRX. Participants were instructed to indicate when the current stimulus matches the one from *n* steps earlier in the sequence. The load factor *n* was 2 for easier version of the task and 3 for the more difficult version. For each of 2- and 3-back conditions, we presented participants with four blocks of 48 stimulus presentations. As in the CPT-AX task, we reported the percentage of correct trials across 2- and 3-back conditions. Stimulus encoding and response demands were constant across conditions; only requirements to maintain and update increasingly greater amounts of information at higher loads differed. Pseudorandom sequences of single consonants were presented, and participants responded to each stimulus, pressing one button to targets and another one to non-targets. Order of task conditions was randomized across participants. Due to the fact that a significant percentage of patients and controls were not successful at 3-back condition, in the further analysis we include only results based on the performance on 2-back condition of N-back task.

### Statistical analysis

For statistical analysis, we used Statistical Package for Social Sciences (SPSS) computer program version 19.0. Neuropsychological variables were tested for the assumptions of normality (Shapiro-Wilk test) and homogeneity of variances (Levene's test). Due to the small number of the participants, lack of normal distribution and homogeneity of variances of some variables, non-parametric analyses of data were performed. Differences in socio-demographic and clinical data between the groups were compared with chi-square test for categorical data and nonparametric tests for continuous data (Kruskal-Wallis and Mann-Whitney tests). Relations between neuropsychological tests' scores with respect to demographic and clinical variables were tested with Spearman's rank correlations. To determine the degree of association in cognitive performance on WM tests between diagnostic groups, we calculated effect size as follows: *r* = Z/vN, where *r* is effect size, Z is z-score and N is the number of observations (Corder and Foreman, 2009). In order to control for co-varying variables, we have used the non-parametric method—rank analysis of covariance (Quade, 1967) (Supplementary Material 2). All tests were two-tailed using a 0.05 level of significance.

## Results

### Impact of demographic variables on cognitive performance

The demographic characteristics, clinical variables and pharmacological status of HC, BPD(−), BPD(+), SZ at the time of neuropsychological testing are summarized in Tables 1, 2. The groups were homogenous with respect to age, number of years of completed education, estimated premorbid IQ, duration of illness and chlorpromazine (CPZ) equivalent dosage. There were no differences across groups with respect to the severity of apathy or depressive symptoms; however, BPD(+) had more pronounced manic symptoms in comparison with BPD(−). It is worth noting that the premorbid IQ scores and number of years of completed education did not differ across groups. This means that our SZ sample may be less representative to the general SZ population, yet suitable for contrasting cognitive characteristics between groups.

Age, number of years of completed education, premorbid IQ and disease duration were not significantly related to task performance (Table 3). However, the CPZ dosage equivalent was correlated with performance on the more demanding WM tasks: Long-delay CPT-AX (*r* = 0.561, *p* < 0.001) and N-back (*r* = 0.458, *p* < 0.001) tests. Additionally, CPZ dosage equivalent was correlated with negative symptoms (PANSS) (*r* = −0.55, *p* = 0.006) and depressive symptoms (HDRS) (*r* = −0.46, *p* = 0.002).

**Table 3:** **Correlations between demographic and clinical variables with WM tests (Digit span forward, Digits span backward, Short-delay CPT-AX test, Long-delay CPT-AX test and N-back test)**.

	**Working memory tests**				
	**Digit Span Forward**	**Digit Span Backward**	**Short-delay CPT-AX**	**Long-delay CPT-AX**	**N-back**
Age	*r* = 0.04	*r* = −0.03	*r* = 0.06	*r* = 0.01	*r* = −0.05
	*p* = 0.56	*p* = 0.80	*p* = 0.59	*p* = 0.91	*p* = 0.66
Number of completed years of education	*r* = −0.08	*r* = 0.06	*r* = 0.04	*r* = 0.02	*r* = −0.01
	*p* = 0.45	*p* = 0.45	*p* = 0.56	*p* = 0.82	*p* = 0.91
Premorbid IQ	*r* = 0.03	*r* = 0.05	*r* = 0.02	*r* = −0.09	*r* = −0.07
	*p* = 0.79	*p* = 0.64	*p* = 0.82	*p* = 0.41	*p* = 0.54
Disease duration	*r* = 0.04	*r* = −0.08	*r* = −0.06	*r* = −0.14	*r* = 0.17
	*p* = 0.75	*p* = 0.53	*p* = 0.61	*p* = 0.27	*p* = 0.17
CPZ equivalent dosage	*r* = −0.131	*r* = −0.125	*r* = 0.091	***r* = 0.561**	***r* = 0.458**
	*p* = 0.29	*p* = 0.32	*p* = 0.46	***p* < 0.0001**	***p* < 0.0001**

r, Spearman's correlation coefficient, p-value- two-tailed.

Significant correlations (*p* < 0.05) were marked in bold.

### Impact of diagnosis on cognitive performance

**Figure 1:**
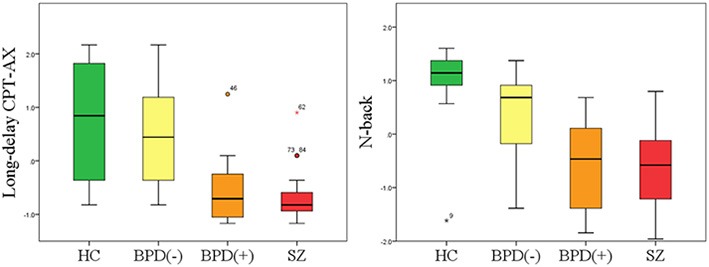
**Neurocognitive performance in HC, BPD(−), BPD(+) and SZ patients on D Long-delay CPT-AX and N-back tasks**. Results are converted to Z-scores for better visualization. Box plots are shown with 95% CI as error bars. Abbreviations: HC, healthy controls; SZ, schizophrenia; BPD(−), bipolar disorder without history of psychosis; BPD(+), bipolar disorder with history of psychosis.

The results of between group comparisons in performance on all WM measures are presented on Figure 1 and in Supplementary Material 3.

Differences in cognitive performance with respect to WM across diagnostic groups depended on the neuropsychological tests applied. Firstly, we analyzed the performance between HC, the combined BPD groups and SZ patients. We found that there were no statistically significant differences in performance on FDS, BDS and Short-delay CPT-AX (*p* > 0.05); however, with respect to Long-delay CPT-AX and N-back tests, SZ patients performed significantly worse than BPD patients (*p* = 0.007 and *p* < 0.001 respectively), and BPD patients performed significantly worse than HC (*p* = 0.002 and *p* = 0.006, respectively).

Further, we compared the cognitive performance between groups by subdividing BPD patients into BPD(−) and BPD(+).There were no significant differences with respect to FDS, BDS and Short-delay CPT-AX between SZ, BPD(+), BPD(−) and HC (*p* > 0.05). However, with respect to Long-delay CPT-AX test, BPD(−) performed equally well as HC (*p* > 0.05). Additionally, BPD(+) and SZ patients performed significantly worse in comparison to HC (*p* < 0.001). With respect to N-back test, BPD(−) patients performed significantly worse than HC (*p* = 0.001), but at the same time significantly better than BPD(+) and SZ patients (*p* < 0.001).

To determine whether performance differences between BPD(+) and BPD(−) in Long-delay CPT-AX and N-back tests were secondary to current symptomatology and/or differences in medication usage, non-parametric analyses of covariance were performed including the following variables: age, gender, number of years of completed education, estimated premorbid IQ, duration of illness, depressive symptoms (HDRS), manic symptoms (YMRS), apathy level (AES) and CPZ equivalent dosage. A significant between-group differences on Long-delay CPT-AX (*p* < 0.0056 after Bonferroni correction) and N-back tests (*p* < 0.0056 after Bonferroni correction) indicated that even with these additional covariates, a history of psychotic symptoms may influence cognitive performance (Supplementary Material 4).

### Impact of clinical ratings on cognitive performance

Correlations between clinical ratings and WM measures indicated that apathy (AES), mania (YMRS), positive and general symptoms (PANSS) were not associated with any neurocognitive outcome on our WM tests (*p* > 0.05). Depression severity (HDRS) was correlated with lower performance on BDS (*r* = −0.463, *p* = 0.023), Long-delay CPT-AX (*r* = −0.535, *p* = 0.007) and N-back (*r* = −0528, *p* = 0.008) among BPD(−) patients. In BPD(+) patients depressive symptoms (HDRS) correlated significantly with Long-delay CPT-AX (*r* = −0.758, *p* < 0.001) and N-back (*r* = −0.584, *p* = 0.009) (Table 4). The severity of negative symptoms (PANSS) was significantly correlated with lower performance on Long-delay CPT-AX (*r* = −0.454, *p* = 0.029) and N-back (*r* = −0.633, *p* = 0.009) among patients with SZ (Table 4).

**Table 4:** **Correlations between clinical ratings and WM tests (Digit span forward, Digits span backward, Short-delay CPT-AX test, Long-delay CPT-AX test and N-back test) with respect to diagnostic groups**.

		**Working memory tests**				
		**Digit span forward**	**Digit span backward**	**Short-delay CPT-AX**	**Long-delay CPT-AX**	**N-back**
BPD(−)	Depressive symptoms (HDRS)	*r* = 0.015	***r* = −0.463**	*r* = −0.536	***r* = −0.535**	***r* = −0.528**
		*p* = 0.944	***p* = 0.023**	*p* = 0.088	***p* = 0.007**	***p* = 0.008**
	Manic symptoms (YMRS)	*r* = −0.232	*r* = −0.144	*r* = −0.053	*r* = −0.244	*r* = 0.370
		*p* = 0.276	*p* = 0.501	*p* = 0.807	*p* = 0.251	*p* = 0.076
BPD(+)	Depressive symptoms (HDRS)	*r* = 0.273	*r* = 0.134	*r* = 0.183	***r* = −0.758**	***r* = −0.584**
		*p* = 0.258	*p* = 0.583	*p* = 0.453	***p*<0.001**	***p* = 0.009**
	Manic symptoms (YMRS)	*r* = 0.105	*r* = 0–0.356	*r* = 0.17	*r* = 0.312	*r* = 0.117
		*p* = 0.668	*p* = 0.134	*p* = 0.944	*p* = 0.251	*p* = 0.632
SZ	Negative symptoms (PANSS)	*r* = 0.012	*r* = 0.158	*r* = −0.076	***r* = −0.454**	***r* = −0.633**
		*p* = 0.958	*p* = 0.472	*p* = 0.732	***p* = 0.029**	***p* = 0.001**

SZ, schizophrenia; BPD(−), bipolar disorder without history of psychosis; BPD(+), bipolar disorder with history of psychosis; PANSS, Positive and Negative Symptoms Scale; YMRS, Young Mania Rating Scale; HDRS, Hamilton Depression Rating Scale; r, Spearman's correlation coefficient, p-value- two-tailed.

Significant correlations (*p* < 0.05) were marked in bold.

## Discussion

### Different domains of WM with respect to psychosis dimension

FDS and BDS are among the most frequently used measures to assess WM in clinical studies of patients with BPD and SZ. In our study, we have shown that FDS and BDS tests did not differentiate HC, BPD(−), BPD(+) and SZ patients, while more demanding tasks such as Long-delay CPT-AX or N-back did. It is in accordance with previous reports showing that FDS and BDS are less sensitive in assessing WM deficits than other WM measures (Perry et al., 2001; Egeland et al., 2003; Haatveit et al., 2010). Additionally, it has been shown that different WM tests require varying amounts of executive function and research using factor analytic approach demonstrated that FDS and BDS tasks load together to form a factor separate from other tests with higher central executive WM demands, such as N-back task (Allen et al., 2010). Farkas et al. (2008) found that schizophrenia patients with severe negative symptoms are more impaired at rule learning tasks than schizophrenia patients with milder negative symptoms.

To the best of our knowledge, this is the first study using tasks measuring the performance on different WM domains such as: the maintenance of information over a variable delay (delay effect), the number of items maintained in WM (load effect), and manipulation of items in WM (manipulation effect). Based on our results, it seems that the effect of psychosis may not be related to manipulation of information in WM, since there was no difference in performance on FDS and BDS tasks. However, a history of psychotic symptoms may influence cognitive performance with respect to WM delay and load effects as measured by Long-delay CPT-AX and N-back tests, respectively.

### Comparison of cognitive performance on short-delay CPT-AX and long-delay CPT-AX tests

In our study, the difficulty level of CPT-AX task was manipulated by prolonging delays between stimuli, and thus increasing the WM load. Longer delays resulted in greater WM deficits in SZ and BPD(+) in comparison with BPD(−) and HC. This may imply that the likelihood of disrupting mental representations increases with time among persons vulnerable to such interference. Our results are in accordance with previous reports showing that an increase in cue-target delay enables researchers to diagnose the selective WM deficit, namely “maintenance difficulty.” This difficulty in maintaining information in WM and using it to produce appropriate response has so far been described in SZ patients (Servan-Schreiber et al., 1996; Lee and Park, 2006) However, the results of our study suggest that it may also be specific to BPD(+) or other groups of patients from psychosis spectrum as WM deficits have been reported in first-episode psychosis patients (Gooding and Tallent, 2004), individuals genetically at risk for schizophrenia (Horan et al., 2008), prodromal and high-risk subjects (Smith et al., 2006; Kelleher et al., 2013), as well as individuals reporting psychotic-like experiences (Laurens et al., 2008).

### Comparison of cognitive performance between BPD(+) and BPD(−) patients

In our study, we have found that there are no differences in performance between BPD(+) and BPD(−) patients with respect to some WM tests (FDS, BDS, Short-delay CPT-AX). With regard to more demanding WM tests (Long-delay CPT-AX and N-back), we were able to observe the difference in the level of cognitive functioning between BPD(+) and BPD(−). This difference prevailed even after controlling for age, gender, number of years of completed education, estimated premorbid IQ, duration of illness, clinical symptoms and CPZ equivalent dosage.

Our results are in line with previous reports showing no significant differences in performance between BPD(+) and BPD(−) on FDS (Glahn et al., 2007; Selva et al., 2007; Martinez-Aran et al., 2008; Savitz et al., 2009; Allen et al., 2010; Brissos et al., 2011) and BDS (Glahn et al., 2007; Selva et al., 2007; Martinez-Aran et al., 2008; Savitz et al., 2009; Allen et al., 2010; Brissos et al., 2011). However, some authors were able to observe differences in cognitive performance between those two groups of patients on these tests (Glahn et al., 2006; Simonsen et al., 2011).

There are few studies showing that WM deficits are indeed associated with the history of psychosis in BPD patients. It has been found that there are significant differences in performance between BPD(+) and BPD(−) with regard to Spatial Delayed Response Task (SDRT) (Glahn et al., 2006, 2007) and Working Memory-Mental Arithmetic Test 2-back (WM-MA) (Simonsen et al., 2011). To the best of our knowledge, our study is the first report showing differences in performance between BPD(+) and BPD(−) with respect to Long-delay CPT-AX and N-back tests, while additionally controlling for demographic and clinical parameters.

Our findings support the notion that the expression of psychotic symptoms may represent a meaningful distinction within the BPD construct. It has also been shown that patients with a history of psychosis, regardless of a diagnosis, are impaired on a variety of cognitive measures, such as executive functioning, verbal learning and memory, verbal fluency, control inference (Glahn et al., 2007; Martinez-Aran et al., 2008; Simonsen et al., 2011; Udal et al., 2012) and additionally, psychotic features in mood disorders have validity in terms of prognosis, treatment response and family history for psychiatric illness (Mazzarini et al., 2010; Souery et al., 2011; Schultze-Lutter et al., 2012). It has also been shown that psychotic symptoms in mood disorders are associated with higher number of hospitalizations (Jager et al., 2005), poorer response to medications (Coryell et al., 1984), increased recurrence (Tohen et al., 2003), greater symptom severity worse short- and long-term outcome (Coryell et al., 2001), longer duration of recovery (Geller et al., 2002) and overall greater functional impairment (Haro et al., 2006). Moreover, executive dysfunction in BPD patients was reported to be related to a history of psychosis in their families (Tabares-Seisdedos et al., 2003).

Future longitudinal research should distinguish whether psychosis is a marker of a more serious developmental subtype of BPD with more pronounced cognitive deficits or a consequence of psychotic episodes in BPD (Udal et al., 2012). Reports of normal pre-morbid cognitive development in BPD support the latter suggestion (Quackenbush et al., 1996; Lewandowski et al., 2011), whereas the results of studies investigating cognitive deficits in two offspring studies indicate the presence of a more serious neurodevelopmental subtype of BPD (Meyer et al., 2004; Maziade et al., 2009). If cognitive deficits are the consequence of psychotic episodes, it emphasizes the importance of identification and treatment of early-onset BPD, in light of the poor prognosis (Bonnín et al., 2010; Torres et al., 2010).

### Comparison of cognitive performance between SZ and BPD(+) patients

In our study, we observed the same level of functioning among SZ and BPD(+) across all cognitive tasks that we applied (FDS, BDS, Short-delay CPT-AX, Long-delay CPT-AX and N-back). Some studies show that SZ patients exhibit more severe WM-related dysfunctions than BPD participants (Hamilton et al., 2009); however, this result might be due to the fact that BPD patients have not been divided into BPD(+) and BPD(−) subgroups. In studies testing performance in BPD(+) and BPD(−) groups separately, it has been shown that BPD(+) group is cognitively closer to the SZ spectrum disorders, than to the BPD(−) group (Simonsen et al., 2011; Ivleva et al., 2012).

Recent evidence indicates common genetic, neurobiological, psychopharmacological and neurocognitive aspects of schizophrenia and psychotic affective disorders. Existing research supports the conceptualization of SZ and BPD as a “psychosis continuum,” that suggests that these two psychotic disorders arise from common neurobiological processes (Ivleva et al., 2010). It has been suggested that BPD(+) sub-phenotype may be a clinical manifestation of gene expression pattern that is common for BPD and SZ. It has been shown that the relation between dopaminergic neurotransmission and psychosis is not unique to SZ but is also observed in BPD(+) (Benedetti et al., 2010). This implies the existence of biological markers associated with the propensity to develop psychosis regardless of a diagnosis of SZ or BPD.

### Cognitive performance of BPD(−) and BPD(+) patients with respect to affective symptoms

In our study, we have shown that depressive symptoms have influence on WM performance in BPD patients. In BPD(+) patients, affective symptoms were associated with performance on Long-delay CPT-AX and N-back tests, while in BPD(+) affective symptoms were also correlated with BDS performance. Our study confirms, that in BPD patients there are state-dependent differences in WM performance that are salient in more demanding WM tasks, which is in line with some studies comparing cognitive performance between manic, depressed and euthymic states in BPD across different cognitive tasks (Martinez-Aran et al., 2004; Rosa et al., 2010). BPD patients have also been found to demonstrate a significant association between WM performance and mood scores (Thermenos et al., 2011), as well as with functional activity changes and morphometric abnormalities of fronto-limbic-striatal gray matter regions implicated in mood regulation (Zanetti et al., 2009; Thermenos et al., 2011). It has been suggested that the activity in WM circuits is affected by the activity in mood regulation circuits, even in euthymic BPD patients, as well as in their first-degree relatives (Thermenos et al., 2011). Neuroimaging studies that are in accordance with our results show that bipolar depression is associated with changes in prefrontal cortex activity during performance of cognitive tasks, especially WM tasks (Townsend et al., 2010). Additionally, reduced activation in the prefrontal cortex has been shown to be inversely correlated with the severity of depressive symptoms (Fernandez-Corcuera et al., 2013).

### Cognitive performance of SZ patients with respect to clinical symptoms

In our study, we observed no association between WM performance with positive symptoms, general symptoms, or apathy in SZ patients. However, there was an inverse correlation between negative symptoms and cognitive performance observed only in more demanding WM tasks with higher central executive WM demands (Long-delay CPT-AX and N-back), but not in less sensitive WM measures (FDS, BDS and Short-delay CPT-AX). This inverse correlation is in accordance with the vast majority of studies repeatedly showing that SZ patients with the most prominent negative symptoms present the greatest cognitive impairment (Brazo et al., 2002; Rodriguez-Sanchez et al., 2008; Dominguez Mde et al., 2009; Sanz et al., 2012), and more specifically also WM deficits (Schmidt-Hansen and Honey, 2009; Barr et al., 2010; Chan et al., 2010). Similarly, studies investigating the relationship between clinical symptoms and cognitive profiles in first-degree relatives of patients with SZ show that neuropsychological deficits partially mediate the increase in negative symptoms (Delawalla et al., 2006; Scala et al., 2012). The relationship between negative symptoms and impaired WM performance converge with brain imaging studies showing that negative symptoms are associated with structural abnormalities of the frontal cortex (Wible et al., 2001).

In our study, we observed the lack of association between WM performance in SZ patients with positive and general symptoms as well as apathy in the presence of a correlation with negative symptoms. These findings may be attributed to the fact that while negative symptomatology is believed to result from decreased dopaminergic activity in the prefrontal cortex directly involved in WM (Okubo et al., 1997; Heckers et al., 1999; Monteleone et al., 2002). Positive and general symptoms as well as apathy are associated with possibly altered activity in the areas less crucial to WM performance, such as the hippocampus (Krieckhaus et al., 1992; Tamminga et al., 2010) and the basal ganglia (Pankow et al., 2012; Khadka et al., 2013; Sorg et al., 2013).

Our study has some limitations that should be discussed. Due to time constraints and the use of many WM tasks, we could not test the effects of SZ and BPD on other cognitive domains, such as executive functions, attention, or learning. Future research should investigate how these disorders affect executive functioning as well as how they influence WM performance. Moreover, in our study we included BPD patients, who were not euthymic and some extent of cognitive dysfunction can be attributed to affective symptoms. However, manic symptoms did not correlate with WM performance and our overall findings are in agreement with previous studies performed on euthymic BPD patients with and without a history of psychosis (Glahn et al., 2006; Simonsen et al., 2011). Additionally, it should also be noted that the severity of depressive symptoms assessed in HDRS correlated significantly with the performance on Long-delay CPT-AX and N-back tasks both in BPD(+) and BPD(−) patients. However, these subgroups of patients differed significantly with respect to the severity of WM impairments evaluated using these tasks. Therefore, between group differences in WM performance can be attributed to a history of psychosis in BPD patients. Other limitations include small sample size of the groups and the lack of assessment with all psychopathology scales across all patients' groups. It has to be noted that we did not apply PANSS scale to assess current symptomatology of BPD(+) patients. This would allow us to analyze the influence of the severity of current symptoms on cognitive functions in this subgroup of patients. However, it should be noted that PANSS scale was primarily developed for the examination of SZ patients. Moreover, the lack of inclusion of patients with schizoaffective disorder might be also perceived as a limitation since such subgroup of subjects would enable more comprehensive insight into the psychosis continuum concept. Additionally, applying a single measure of psychotic symptoms severity to all participants, or at least all patients, would allow more detailed exploration of psychosis with respect to a categorical diagnostic system.

In summary, measuring different aspects of WM allows observation of between group differences in cognitive performance among BPD and SZ patients. These differences may depend on the patients' history of psychosis rather than a categorical diagnostic group defined by current diagnostic systems.

### Conflict of interest statement

The authors declare that the research was conducted in the absence of any commercial or financial relationships that could be construed as a potential conflict of interest.
