# Supplementary material for: Impairments of working memory in schizophrenia and bipolar disorder: the effect of history of psychotic symptoms and different aspects of cognitive task demands
Source: Front Behav Neurosci. 2014 Nov 28;8:416. doi: 10.3389/fnbeh.2014.00416 (PMC4246891; doi:10.3389/fnbeh.2014.00416)
Supplement: Supplementary file 1 [file DataSheet1.DOCX]

Supplementary material 1. Raw data of the study.

| **Date of receiving subject's data** | **Subj ID** | **Group** | **Male/Female** | **Age** | **Education** | **Bipolar disorder (I or II)** | **Disease Duration** | **PANSS-Positive** | **PANSS-Negative** | **PANSS-General** | **Mania** | **Depression** | **Apathy** | **NAART** | **Medication** | **Chlorp equivalent dose** | **forward Digit Span** | **backward digit span** | **WM-short delay (Correct)** | **WM-LongDelay (correct)** | **n-back (correct %)** |
| --- | --- | --- | --- | --- | --- | --- | --- | --- | --- | --- | --- | --- | --- | --- | --- | --- | --- | --- | --- | --- | --- |
| 5-Nov-12 | KS16 | Nonpsychotic BD | M | 36 | 14 | II | 15 |  |  |  | 18 | 24 | 27 | 54 | Risperidone | 133 | 10 | 7 | 85 | 67 | 77 |
| 5-Nov-12 | AT215 | Nonpsychotic BD | f | 31 | 15 | II | 12 |  |  |  | 16 | 20 | 34 | 20 | Clozapine +other antipsych meds | 583 | 7 | 9 | 87 | 89 | 89 |
| 5-Nov-12 | AT216 | Nonpsychotic BD | M | 43 | 13 | I | 25 |  |  |  | 22 | 28 | 30 | 41 | Clozapine +other antipsych meds | 536 | 6 | 6 | 82 | 80 | 69 |
| 5-Nov-12 | AT218 | Nonpsychotic BD | M | 39 | 12 | II | 22 |  |  |  | 21 | 27 | 26 | 45 | Chlorpromazine +other antipsych meds | 599 | 9 | 4 | 71 | 65 | 87 |
| 5-Nov-12 | DM31 | Nonpsychotic BD | f | 39 | 5 | I | 15 |  |  |  | 25 | 20 | 35 | 44 | Haloperidol +other antipsych meds | 632 | 5 | 9 | 78 | 74 | 87 |
| 7-Oct-11 | DM32 | Nonpsychotic BD | M | 41 | 13 | II | 15 |  |  |  | 19 | 23 | 33 | 32 | Risperidone | 350 | 11 | 4 | 86 | 77 | 85 |
| 7-Oct-11 | DM34 | Nonpsychotic BD | f | 41 | 14 | II | 18 |  |  |  | 17 | 20 | 30 | 21 | Chlorpromazine | 642 | 6 | 4 | 87 | 85 | 86 |
| 7-Oct-11 | AT220 | Nonpsychotic BD | M | 40 | 12 | II | 17 |  |  |  | 14 | 22 | 38 | 21 | Risperidone | 105 | 9 | 9 | 84 | 63 | 65 |
| 7-Oct-11 | AT221 | Nonpsychotic BD | f | 44 | 11 | II | 17 |  |  |  | 19 | 22 | 49 | 30 | Risperidone | 247 | 10 | 4 | 84 | 71 | 82 |
| 7-Oct-11 | AT222 | Nonpsychotic BD | M | 43 | 5 | II | 15 |  |  |  | 18 | 24 | 42 | 31 | Clozapine | 248 | 8 | 8 | 62 | 71 | 71 |
| 2-Mar-12 | DM36 | Nonpsychotic BD | f | 49 | 13 | I | 26 |  |  |  | 22 | 20 | 45 | 43 | Chlorpromazine | 601 | 7 | 9 | 83 | 74 | 85 |
| 2-Mar-12 | AT223 | Nonpsychotic BD | M | 39 | 13 | II | 15 |  |  |  | 19 | 20 | 33 | 4 | Risperidone | 529 | 10 | 9 | 81 | 78 | 79 |
| 2-Mar-12 | AM56 | Nonpsychotic BD | f | 38 | 14 | II | 17 |  |  |  | 13 | 28 | 41 | 43 | Clozapine +other antipsych meds | 107 | 7 | 8 | 80 | 65 | 73 |
| 2-Mar-12 | AM57 | Nonpsychotic BD | M | 41 | 12 | II | 14 |  |  |  | 19 | 25 | 25 | 36 | Haloperidol | 174 | 6 | 4 | 80 | 67 | 74 |
| 2-Mar-12 | AM58 | Nonpsychotic BD | M | 42 | 14 | I | 18 |  |  |  | 21 | 23 | 31 | 31 | Risperidone | 463 | 5 | 4 | 79 | 81 | 84 |
| 17-Sep-11 | AM59 | Nonpsychotic BD | M | 44 | 14 | I | 15 |  |  |  | 25 | 20 | 46 | 36 | Haloperidol | 481 | 11 | 9 | 84 | 82 | 85 |
| 17-Sep-11 | AM61 | Nonpsychotic BD | f | 49 | 12 | II | 21 |  |  |  | 11 | 23 | 48 | 35 | Haloperidol | 379 | 10 | 9 | 64 | 65 | 74 |
| 8-Mar-12 | OL03 | Nonpsychotic BD | M | 51 | 13 | II | 26 |  |  |  | 16 | 21 | 36 | 34 | Chlorpromazine +other antipsych meds | 488 | 10 | 8 | 71 | 77 | 83 |
| 6-Feb-12 | OL04 | Nonpsychotic BD | f | 45 | 13 | I | 17 |  |  |  | 24 | 22 | 29 | 38 | Chlorpromazine | 411 | 9 | 8 | 72 | 69 | 83 |
| 6-Feb-12 | Ol05 | Nonpsychotic BD | f | 53 | 12 | II | 19 |  |  |  | 16 | 30 | 30 | 31 | Risperidone | 131 | 11 | 7 | 61 | 65 | 78 |
| 3-Mar-12 | OKL34 | Nonpsychotic BD | M | 49 | 16 | II | 25 |  |  |  | 15 | 24 | 48 | 38 | Risperidone +other antipsych meds | 588 | 9 | 8 | 84 | 84 | 79 |
| 23-Mar-12 | OKL35 | Nonpsychotic BD | f | 46 | 6 | II | 17 |  |  |  | 11 | 22 | 37 | 31 | Clozapine | 204 | 10 | 8 | 80 | 76 | 85 |
| 5-Nov-12 | AM62 | Nonpsychotic BD | M | 43 | 8 | I | 16 |  |  |  | 22 | 21 | 28 | 41 | Haloperidol | 590 | 9 | 6 | 63 | 84 | 86 |
| 12-Aug-11 | DH81 | Nonpsychotic BD | M | 48 | 12 | II | 22 |  |  |  | 19 | 27 | 48 | 38 | Risperidone | 464 | 9 | 7 | 71 | 70 | 85 |
| 12-Mar-11 | KS28 | Psychotic BD | M | 47 | 6 | I | 25 |  |  |  | 26 | 27 | 25 | 34 | Risperidone | 341 | 11 | 5 | 71 | 60 | 72 |
| 12-Mar-11 | DH75 | Psychotic BD | f | 43 | 15 | I | 19 |  |  |  | 21 | 24 | 39 | 43 | Haloperidol | 248 | 6 | 9 | 78 | 61 | 78 |
| 12-Mar-11 | DH76 | Psychotic BD | M | 40 | 13 | I | 19 |  |  |  | 27 | 24 | 49 | 39 | Risperidone | 256 | 10 | 9 | 67 | 69 | 79 |
| 12-Mar-11 | AT191 | Psychotic BD | M | 47 | 12 | I | 19 |  |  |  | 28 | 17 | 33 | 38 | Chlorpromazine +other antipsych meds | 691 | 8 | 4 | 67 | 81 | 83 |
| 12-Apr-11 | AT334 | Psychotic BD | f | 38 | 12 | I | 9 |  |  |  | 27 | 24 | 35 | 31 | Clozapine | 202 | 9 | 9 | 79 | 66 | 76 |
| 20-Jun-11 | AT335 | Psychotic BD | M | 47 | 15 | I | 20 |  |  |  | 26 | 19 | 36 | 43 | Chlorpromazine | 451 | 8 | 8 | 87 | 65 | 65 |
| 20-Jun-11 | AT336 | Psychotic BD | f | 48 | 12 | I | 26 |  |  |  | 28 | 25 | 42 | 32 | Clozapine | 455 | 9 | 4 | 77 | 64 | 65 |
| 20-Jun-11 | KS29 | Psychotic BD | M | 43 | 12 | I | 19 |  |  |  | 28 | 24 | 44 | 38 | Clozapine | 228 | 8 | 5 | 79 | 61 | 62 |
| 20-Jun-11 | KS30 | Psychotic BD | f | 49 | 13 | II | 27 |  |  |  | 15 | 28 | 26 | 31 | Chlorpromazine | 322 | 8 | 8 | 78 | 62 | 61 |
| 12-Aug-11 | KS31 | Psychotic BD | M | 46 | 5 | I | 21 |  |  |  | 24 | 20 | 42 | 28 | Clozapine | 441 | 6 | 7 | 76 | 70 | 79 |
| 12-Aug-11 | KS32 | Psychotic BD | f | 38 | 12 | II | 11 |  |  |  | 16 | 26 | 25 | 40 | Chlorpromazine | 358 | 7 | 5 | 79 | 61 | 71 |
| 12-Aug-11 | KS33 | Psychotic BD | M | 45 | 7 | II | 21 |  |  |  | 14 | 26 | 29 | 6 | Clozapine | 430 | 10 | 6 | 72 | 60 | 74 |
| 12-Aug-11 | KS36 | Psychotic BD | M | 47 | 12 | I | 19 |  |  |  | 28 | 24 | 44 | 40 | Clozapine | 440 | 5 | 5 | 83 | 63 | 75 |
| 12-Aug-11 | KS38 | Psychotic BD | f | 46 | 14 | I | 16 |  |  |  | 26 | 24 | 42 | 45 | Chlorpromazine | 339 | 9 | 5 | 81 | 65 | 71 |
| 12-Aug-11 | KS39 | Psychotic BD | M | 42 | 15 | I | 19 |  |  |  | 26 | 24 | 38 | 32 | Clozapine | 426 | 8 | 5 | 62 | 67 | 78 |
| 12-Aug-11 | LT05 | Psychotic BD | M | 54 | 13 | I | 24 |  |  |  | 21 | 26 | 47 | 52 | Chlorpromazine | 311 | 7 | 9 | 86 | 61 | 61 |
| 12-Aug-11 | LT06 | Psychotic BD | f | 43 | 5 | I | 14 |  |  |  | 23 | 23 | 27 | 13 | Clozapine | 565 | 10 | 6 | 82 | 70 | 78 |
| 12-Aug-11 | LT07 | Psychotic BD | M | 45 | 14 | I | 18 |  |  |  | 25 | 19 | 31 | 17 | Clozapine | 585 | 7 | 6 | 62 | 71 | 73 |
| 12-Aug-11 | KS41 | Psychotic BD | M | 40 | 14 | I | 22 |  |  |  | 27 | 27 | 36 | 45 | Clozapine | 435 | 9 | 9 | 89 | 64 | 64 |
| 12-Aug-11 | KS42 | Schizo | M | 52 | 14 |  | 21 | 22 | 16 | 21 |  |  | 26 | 43 | Haloperidol +other antipsych meds | 557 | 8 | 5 | 84 | 78 | 76 |
| 13-Oct-11 | KS43 | Schizo | M | 47 | 14 |  | 19 | 11 | 26 | 32 |  |  | 31 | 50 | Haloperidol | 444 | 5 | 8 | 82 | 63 | 66 |
| 13-Oct-11 | KS44 | Schizo | f | 39 | 13 |  | 15 | 23 | 15 | 19 |  |  | 46 | 30 | Clozapine | 446 | 9 | 7 | 79 | 65 | 67 |
| 17-Sep-12 | AT412 | Schizo | M | 42 | 13 |  | 18 | 19 | 18 | 22 |  |  | 41 | 25 | Chlorpromazine +other antipsych meds | 446 | 9 | 9 | 85 | 64 | 76 |
| 13-Oct-11 | KS45 | Schizo | M | 46 | 12 |  | 21 | 15 | 21 | 21 |  |  | 36 | 29 | Chlorpromazine | 488 | 7 | 5 | 81 | 62 | 72 |
| 13-Oct-11 | AT414 | Schizo | f | 42 | 13 |  | 21 | 21 | 21 | 32 |  |  | 42 | 33 | Chlorpromazine | 231 | 5 | 6 | 76 | 65 | 69 |
| 3-Jul-12 | GT212 | Schizo | M | 41 | 14 |  | 17 | 15 | 17 | 23 |  |  | 49 | 33 | Clozapine +other antipsych meds | 457 | 8 | 5 | 76 | 64 | 75 |
| 7-May-12 | FT213 | Schizo | f | 45 | 6 |  | 21 | 22 | 21 | 31 |  |  | 38 | 34 | Clozapine | 336 | 5 | 4 | 78 | 60 | 69 |
| 13-Oct-11 | AT415 | Schizo | M | 38 | 12 |  | 16 | 16 | 22 | 31 |  |  | 36 | 34 | Clozapine | 328 | 9 | 6 | 75 | 64 | 67 |
| 12-Apr-11 | AT11 | Schizo | f | 49 | 15 |  | 22 | 17 | 29 | 26 |  |  | 27 | 41 | Chlorpromazine | 325 | 8 | 7 | 79 | 61 | 71 |
| 12-Apr-11 | AT12 | Schizo | M | 47 | 5 |  | 19 | 13 | 23 | 16 |  |  | 43 | 39 | Clozapine | 437 | 10 | 5 | 84 | 67 | 63 |
| 12-Aug-11 | AT13 | Schizo | M | 33 | 12 |  | 11 | 11 | 12 | 32 |  |  | 44 | 31 | Chlorpromazine +other antipsych meds | 561 | 5 | 6 | 88 | 71 | 75 |
| 5-Nov-12 | AT14 | Schizo | f | 41 | 13 |  | 23 | 13 | 25 | 18 |  |  | 38 | 44 | Clozapine | 158 | 5 | 5 | 78 | 62 | 63 |
| 5-Nov-12 | AT16 | Schizo | M | 37 | 14 |  | 12 | 21 | 19 | 22 |  |  | 29 | 28 | Clozapine | 310 | 7 | 4 | 88 | 62 | 64 |
| 5-Nov-12 | AT17 | Schizo | f | 42 | 6 |  | 17 | 18 | 17 | 32 |  |  | 46 | 38 | Risperidone | 397 | 8 | 4 | 84 | 63 | 78 |
| 5-Nov-12 | AT19 | Schizo | M | 53 | 14 |  | 24 | 19 | 15 | 19 |  |  | 25 | 28 | Clozapine | 456 | 8 | 5 | 78 | 71 | 84 |
| 5-Nov-12 | TR42 | Schizo | M | 41 | 15 |  | 21 | 20 | 23 | 23 |  |  | 34 | 36 | Clozapine | 321 | 9 | 8 | 83 | 62 | 61 |
| 5-Nov-12 | TR43 | Schizo | f | 50 | 7 |  | 26 | 12 | 15 | 25 |  |  | 47 | 43 | Risperidone | 276 | 10 | 7 | 77 | 61 | 73 |
| 5-Nov-12 | TR45 | Schizo | M | 42 | 12 |  | 17 | 17 | 16 | 21 |  |  | 27 | 37 | Clozapine | 361 | 7 | 6 | 80 | 62 | 74 |
| 5-Nov-12 | VL44 | Schizo | f | 40 | 12 |  | 13 | 11 | 21 | 19 |  |  | 42 | 39 | Clozapine | 217 | 6 | 9 | 62 | 60 | 76 |
| 5-Nov-12 | TT09 | Schizo | M | 31 | 12 |  | 9 | 21 | 21 | 26 |  |  | 33 | 35 | Haloperidol +other antipsych meds | 323 | 6 | 9 | 63 | 61 | 79 |
| 5-Nov-12 | KO12 | Schizo | M | 39 | 13 |  | 11 | 14 | 23 | 27 |  |  | 29 | 39 | Haloperidol | 207 | 11 | 8 | 71 | 62 | 60 |
| 9-Nov-12 | EE39 | Schizo | f | 31 | 15 |  | 9 | 13 | 9 | 25 |  |  |  |  | Clozapine | 497 | 6 | 7 | 64 | 71 | 78 |
| 9-Nov-12 | TR46 | HC | M | 29 | 14 | NA | NA |  |  |  |  |  | 46 | 42 | NA | NA | 11 | 4 | 82 | 67 | 86 |
| 9-Nov-12 | KL112 | HC | M | 44 | 5 | NA | NA |  |  |  |  |  | 36 | 40 | NA | NA | 8 | 8 | 84 | 89 | 89 |
| 13-Oct-11 | KL113 | HC | f | 42 | 13 | NA | NA |  |  |  |  |  | 45 | 33 | NA | NA | 9 | 5 | 82 | 88 | 88 |
| 13-Oct-11 | KL114 | HC | M | 44 | 15 | NA | NA |  |  |  |  |  | 29 | 32 | NA | NA | 8 | 7 | 85 | 81 | 84 |
| 13-Oct-11 | KL116 | HC | f | 38 | 14 | NA | NA |  |  |  |  |  | 41 | 38 | NA | NA | 5 | 6 | 70 | 86 | 88 |
| 13-Oct-11 | KL117 | HC | M | 46 | 13 | NA | NA |  |  |  |  |  | 43 | 19 | NA | NA | 6 | 4 | 84 | 72 | 87 |
| 13-Oct-11 | AT20 | HC | M | 41 | 12 | NA | NA |  |  |  |  |  | 38 | 39 | NA | NA | 6 | 7 | 63 | 65 | 89 |
| 13-Oct-11 | AT21 | HC | M | 52 | 13 | NA | NA |  |  |  |  |  | 44 | 35 | NA | NA | 9 | 6 | 87 | 85 | 88 |
| 13-Oct-11 | AT22 | HC | f | 53 | 12 | NA | NA |  |  |  |  |  | 45 | 23 | NA | NA | 5 | 9 | 82 | 63 | 63 |
| 13-Oct-11 | KL118 | HC | M | 43 | 14 | NA | NA |  |  |  |  |  | 39 | 30 | NA | NA | 9 | 5 | 62 | 76 | 86 |
| 13-Oct-11 | KL119 | HC | M | 35 | 6 | NA | NA |  |  |  |  |  | 45 | 49 | NA | NA | 9 | 8 | 67 | 73 | 85 |
| 13-Oct-11 | KL121 | HC | M | 46 | 12 | NA | NA |  |  |  |  |  | 47 | 32 | NA | NA | 6 | 6 | 81 | 64 | 85 |
| 13-Oct-11 | DM07 | HC | f | 53 | 13 | NA | NA |  |  |  |  |  | 31 | 23 | NA | NA | 5 | 9 | 83 | 67 | 89 |
| 13-Oct-11 | DM08 | HC | M | 48 | 13 | NA | NA |  |  |  |  |  | 41 | 44 | NA | NA | 9 | 5 | 80 | 83 | 82 |
| 3/3/2012 | GR101 | HC | f | 57 | 12 | NA | NA |  |  |  |  |  | 32 | 41 | NA | NA | 8 | 6 | 84 | 79 | 87 |
| 5/3/2012 | GR102 | HC | M | 47 | 13 | NA | NA |  |  |  |  |  | 40 | 43 | NA | NA | 10 | 5 | 87 | 89 | 84 |
| 6/4/2012 | GR104 | HC | f | 43 | 14 | NA | NA |  |  |  |  |  | 37 | 41 | NA | NA | 10 | 7 | 65 | 76 | 89 |
| 2/3/2012 | DF12 | HC | M | 37 | 7 | NA | NA |  |  |  |  |  | 40 | 30 | NA | NA | 9 | 8 | 82 | 89 | 91 |
