# Supplementary material for: Impairments of working memory in schizophrenia and bipolar disorder: the effect of history of psychotic symptoms and different aspects of cognitive task demands
Source: Front Behav Neurosci. 2014 Nov 28;8:416. doi: 10.3389/fnbeh.2014.00416 (PMC4246891; doi:10.3389/fnbeh.2014.00416)
Supplement: Supplementary file 2 [file DataSheet2.DOCX]

**Supplementary material 2.** Detailed statistical analysis.

Non-parametric analysis of covariance was performed using the method described by Quade (1967). We undertook the following steps in calculating analysis of covariance in our study:

1) ranking the dependent variable and any covariates

2) performing linear regression of the ranks of the dependent variable on the ranks of the covariates

3) performing a one-way analysis of variance (ANOVA), using the residuals from the regression in the prior step as the dependent variable, and the grouping variable as the factor. The F test resulting from this ANOVA is the F statistic used by Quade (1967).

In order to calculate non-parametric analysis of covariance, we have written the following program in the MathWorks software:

function naoctool(x,y,group)

ix = ~isnan(x);

iy = ~isnan(y);

idx = ix .* iy;

xx = [];

yy = [];

gg = [];

for i = 1:length(idx)

if idx(i) == 1;

xx = [xx; x(i)];

yy = [yy; y(i)];

gg = [gg; group(i)];

end

end

x = xx;

y = yy;

group = gg;

xranks = tiedrank(x);

yranks = tiedrank(y);

X = [ones(size(xranks)) xranks];

[b,bint,r] = regress(yranks,X);

b

bint

Xaxis=[min(xranks); max(xranks)];

plot(xranks,yranks,'ko'); hold on

plot(Xaxis,b(2)*Xaxis+b(1),'k');

xlabel('xranks');

ylabel('yranks');

p = anova1(r,group)

end

load must;

descR={'Age' 'Apathy' 'Chlorpromazine equivalent' 'Depression' 'Durartion' 'Education' 'Mania' 'NAART' 'WMlong' 'WMshort' 'backwardDigit' 'forwardDigit' 'nBack'};

Y=[ Age Apathy Chlorpromazine, Depression Duration Education Mania NAART WMlong WMsh backwardDigit forwardDigit nBack];

Gname={'HC' 'NonpsychoticBP' 'PsychoticBP' 'Schizophrenia'};

k=1;

for i=1:1

for j=i+1:size(Y,2)

figure(k)

k = k + 1;

aoctool(Y(:,i),Y(:,j),Group, 0.05, descR{i},descR{j});

end

end

**References**

Quade D (1967) Rank analysis of covariance. Journal of the American Statistical Association 62:1187-1200.
