# Supplementary material for: Impairments of working memory in schizophrenia and bipolar disorder: the effect of history of psychotic symptoms and different aspects of cognitive task demands
Source: Front Behav Neurosci. 2014 Nov 28;8:416. doi: 10.3389/fnbeh.2014.00416 (PMC4246891; doi:10.3389/fnbeh.2014.00416)
Supplement: Supplementary file 3 [file DataSheet3.DOCX]

**Supplementary material 3.** Between groups differences of the performance on the WM tests (Digit Span Forward, Digits Span Backward, Short-delay CPT-AX test, Long-delay CPT-AX test and N-back test) with effect size calculated according to ([Corder and Foreman, 2009](#_ENREF_17)).

|  | **Working Memory Tests** | | | | |
| --- | --- | --- | --- | --- | --- |
|  | **Manipulation of information in WM** | | **WM delay effect** | | **WM load** |
|  | Digit Span Forward | Digit Span Backward | Short-delay CPT-AX | Long-delay CPT-AX | N-back |
| HC(-) vs. BPD(-) | M_HC_=7.89. vs.  M_BPD(-)_=.7.69 (U=169.5, ns,  r= -0.19) | M_HC_=6.39 vs.  M_BPD(-)_=7.00 (U=169.5, ns,  r= -0.18) | M_HC_=78.33 vs. M_BPD(-)_=0.77.46 (U=197, ns,  r= -0.07) | M_HC_=77.33 vs.  M_BPD(-)_=74.12 (U=172.0, ns,  r= -0.17) | **M_HC_=85.56 vs.**  **M_BPD(-)_=80.46 (U=90.5, p=0.001**  **r= -0.49)** |
| HC(-) vs. BPD(+) | M_HC_=7.89 vs. M_BPD(+)_= 7.40 (U=162.5, ns,  r= -0.04) | M_HC_=6,39 vs. M_BPD(+)_=6,53 (U=166.5, ns,  r= -0.02) | M_HC_=78.33 vs. M_BPD(+)_=76.58 (U=131.0, ns,  r= -0.04) | **M_HC_=77,33** **vs. M_BPD(+)_=65.32**  **(U=46.0, p=0.000**  **r= -0.62)** | **M_HC_=85,56 .vs. M_BPD(+)_=0.71.84 (U=17.0, p=0.000**  **r= -0.77)** |
| HC(-) vs. SZ | M_HC_=7.89vs. M_SZ_=7.43  (U=174.0, ns,  r= -0.14) | M_HC_=6.39 21,42 vs. M_SZ_=0.6.30 (U=199.5, ns,  r= -0.03) | M_HC_=78.33 vs. M_SZ_=78,04 (U=178.5, ns,  r= -0.12) | **M_HC_=77.33 0. vs. M_SZ_=64.39**  **(U=38.5, p=0.000**  **r= -0.69)** | **M_HC_=85.56 vs. M_SZ_=71.13**  **(U=22.0. p=0.000**  **r= -0.76)** |
| BPD(-) vs. BPD(+) | M_BPD(-)_=7.69 vs. M_BPD(+)_=7.40 0.(U=194.5, ns,  r= -0.13) | M_BPD(-)_=7.00 vs. M_BPD(+)_=6.53 0.(U=203.0, ns,  r= -0.09) | M_BPD(-)_=77.46 vs. M_BPD(+)_=76.58 0.(U=197.5, ns,  r= -0.11) | **M_BPD(-)_=74.12 vs. M_BPD(+)_=65.32 (U=262.5, p=0.000**  **r= -0.58)** | **M_BPD(-)_=80.46 vs. M_BPD(+)_=71,84**  **(U=82.0, p=0.000**  **r= -0.55)** |
| BPD(-) vs. SZ | **M_BPD(-)_=7.69 vs. M_SZ_=7.430.**  **(U=184.0, p=0.047**  **r= -0.29)** | M_BPD(-)_=7.00 .5vs. M_SZ_=6.30  (U=214.5, ns,  r= -0.019) | M_BPD(-)_=77.46 vs. M_SZ_=78.04 (U=267.0, ns,  r= -0.03) | **M_BPD(-)_=74.12**  **vs. M_SZ_=64,.9**  **(U=62.5, p=0.000**  **r= -0.66)** | **M_BPD(-)_=80.46 vs. M_SZ_=71.13**  **(U=87.5, p=0.000**  **r= -0.59)** |
| BPD(+) vs. SZ | M_BPD(+)_=7.40 vs. M_SZ_=7.43  (U=168.0, ns,  r= -0.20) | M_BPD(+)_=6.53 vs. M_SZ_=6,30 0.(U=205.5, ns,  r= -0.05) | M_BPD(-)_=77.46 0.vs. M_SZ_ 78.04 .5(U=194.5, ns,  r= -0.09) | M_BPD(+)_=65.32 vs. M_SZ_=64.39 0.(U=201.0, ns,  r= -0.07) | M_BPD(+)_=22.39 vs. M_SZ_=71.130.  (U=201.5, ns,  r= -0.07) |

Abbreviations: HC, healthy controls; SZ, schizophrenia; BPD (-), bipolar disorder without history of psychosis; BPD (+), bipolar disorder with history of psychosis, M - mean value, U – Mann Whitney, ns- not significant, p value- two-tailed, r- effect size
