# Supplementary material for: Impairments of working memory in schizophrenia and bipolar disorder: the effect of history of psychotic symptoms and different aspects of cognitive task demands
Source: Front Behav Neurosci. 2014 Nov 28;8:416. doi: 10.3389/fnbeh.2014.00416 (PMC4246891; doi:10.3389/fnbeh.2014.00416)
Supplement: Supplementary file 4 [file DataSheet4.DOCX]

**Supplementary material 4.** Differences in performance on WM tasks between BPD(+) and BPD(-) patients co-varying for age, gender, education level, NAART, CPZ equivalent dosage, duration of illness, YMRS, HDRS, AES (p-values of non-parametric analysis of covariance are reported).

|  | Digit Span Forward | Digit Span Backward | Short-delay CPT-AX | Long-delay CPT-AX* | N-back* |
| --- | --- | --- | --- | --- | --- |
| Age | 0.3300 | 0.553 | 0.6037 | 0.00005 | 0.0002 |
| Gender | 0.4026 | 0.5649 | 0.4715 | 0.00003 | 0.0001 |
| Education^a^ | 0.3943 | 0.5459 | 0.4527 | 0.00003 | 0.0001 |
| NAART^b^ | 0.4389 | 0.5154 | 0.4319 | 0.00003 | 0.0001 |
| CPZ^c^ equivalent dosage | 0.3282 | 0.4945 | 0.4597 | 0.000002 | 0.00004 |
| Duration of illness^d^ | 0.4299 | 0.5465 | 0.6087 | 0.0001 | 0.0004 |
| YMRS^e^ | 0.7883 | 0.9955 | 0.6304 | 0.0002 | 0.0009 |
| HDRS^f^ | 0.3663 | 0.6334 | 0.5472 | 0.00001 | 0.0002 |
| AES^g^ | 0.4116 | 0.4946 | 0.4543 | 0.00003 | 0.0001 |

a = number of years of completed education; b = estimate of premorbid IQ measured by North American Adult Reading Test (NAART); c= CPZ – chlorpromazine; d = number of years of illness, e - YMRS - Young Mania Rating Scale; f = HDRS - Hamilton Depression Rating Scale; g = AES - Apathy Evaluation Scale

* p-value significant after Bonferoni correction (p < 0.0056)
